# Professionalism and professional identity in medical students: a cross-sectional mixed-methods analysis of correlation and progression

**DOI:** 10.3389/fmed.2025.1650073

**Published:** 2025-09-15

**Authors:** Munawar Farooq, Muhammad Jawad Hashim, Sultan Alhosani, Amer Mohammed Alalawi, Omar Mohammed Alalawi, Khaled Abdullah Qandilo, Uffaira Hafeez, Arif Alper Cevik

**Affiliations:** ^1^Department of Internal Medicine, Emergency Medicine Section, College of Medicine and Health Sciences, United Arab Emirates University, Al-Ain, United Arab Emirates; ^2^Department of Emergency Medicine, Tawam Hospital, Al-Ain, United Arab Emirates; ^3^Department of Family Medicine, College of Medicine and Health Sciences, United Arab Emirates University, Al-Ain, United Arab Emirates; ^4^Medical Student, College of Medicine and Health Sciences, United Arab Emirates University, Al-Ain, United Arab Emirates; ^5^Research Office, College of Medicine and Health Sciences, United Arab Emirates University, Al-Ain, United Arab Emirates

**Keywords:** medical education, professionalism, professionalism assessment scale, professional identity, empathy

## Abstract

**Introduction:**

Professionalism is a core competency in undergraduate and graduate medical education. Professional identity is the internalization of a community’s norms, which results in thoughts, feelings, and actions that align with that community. Uncertainty exists regarding the interrelated progression of professionalism and professional identity. We aimed to explore the professionalism and professional identity scores, as well as their internal correlation, among medical students across different stages of their education.

**Methods:**

We conducted a cross-sectional, observational, complementary mixed-methods study among medical students across all curriculum stages. Quantitative data were collected using the Professionalism Assessment Scale (PAS) and the Professional Identity Questionnaire (PIQ). Qualitative data from two open-ended questions explored students’ perspectives on learning and assessing professionalism.

**Results:**

One hundred and eight medical students completed the study questionnaires. The reliability coefficient (Cronbach’s alpha) was 0.763 for the PAS questionnaire and 0.767 for the PIQ instrument. PIQ scores had low correlation with empathy, PAS, professional relationships, and responsibility (all *r* values <0.14). Professionalism (PAS, *p* = 0.024), empathy (*p* = 0.009), and professional Relationships (*p* = 0.007) scores were significantly higher among students in pre-clinical compared to those in basic sciences, however, no significant differences were observed in these scores among students in the pre-clinical, early clinical, and late clinical stages. Professional Responsibility (*p* = 0.60) and Professional Identity (*p* = 0.57) scores showed no significant change across all stages.

**Conclusion:**

The study highlights plateaus in professionalism and professional identity scores, with no significant correlation between PIQ and PAS scores. While prior literature often treats the two as overlapping or sequential frameworks, our findings suggest the need for separate, targeted strategies in curriculum design to advance both professionalism and professional identity.

## 1 Introduction

Professionalism is a core competency in both undergraduate and graduate medical education. The definition of professionalism in medicine lacks consensus (1). Three commonly described frameworks for medical professionalism include ethics/virtues, skills/behavior, and professional identity (2). Medical professionalism is integral to enhancing healthcare delivery and improving patient outcomes, making its incorporation into medical education a vital component for training competent healthcare professionals (3). Professional attributes such as integrity, compassion, communication skills, and lifelong learning guide medical students toward becoming healers in healthcare (4). Professional identity refers to the evolving self-concept through which medical students and physicians internalize professional values, attitudes, and behaviors. It is a complex, non-linear process shaped by mentorship, role modeling, ethical codes, and cultural ideals of the “good physician,” and reinforced as learners embody these values and assume their professional roles (5).

To understand how these attributes develop, it is important to consider the theoretical foundations of professionalism and professional identity formation (PIF) which explain how learners internalize values, norms, and roles over time. Social learning theory underscores the importance of role modeling and observation in shaping professional behaviors (6, 7). The theory of communities of practice further posits that identity formation is a social process in which learners transition from peripheral to full participation within professional groups (8, 9).

Teaching medical professionalism ensures the cultivation of a professional identity, with Professional Identity Formation (PIF) being the process by which physicians internalize the values, attitudes, and attributes necessary for their role (10). A well-formed professional identity is required for the successful application of medical skills and knowledge, making it a fundamental goal of medical education (11). Importantly, professionalism and professional identity have been described as bidirectional and mutually reinforcing constructs. Professionalism provides the external behaviors and standards through which identity is expressed, while professional identity underpins and sustains the internalization of those professional values (12).

The literature presents conflicting evidence regarding the trajectory of medical professionalism attributes throughout medical school. Empathy, a fundamental component of medical professionalism, has been reported to decline during medical school (13). In contrast, other studies have found no significant difference in empathy scores across medical school years (14). One study reported an initial decrease in empathy, followed by a plateau and a subsequent increase (15). Similarly, professionalism scores have been reported to decline initially, followed by an increase in some studies, while other studies observed no significant change (16, 17). The relationship between professional identity and progression through medical school remains inconclusive. While some studies report a positive correlation with advancing years of study, others have found no significant association (18, 19). Although prior studies have examined professionalism and professional identity separately, few have investigated both constructs together to determine whether they develop in parallel or along distinct pathways and how they vary across different curricular stages, particularly in Middle Eastern contexts. To address these gaps, this study aimed to assess patterns of professionalism and professional identity among undergraduate medical students across curriculum phases, examine the relationship between these constructs, and explore how students learn about professionalism and their perspectives on its assessment to inform curriculum strategies.

## 2 Materials and methods

### 2.1 Study design

This study employed a cross-sectional, observational, complementary mixed-methods design in which quantitative and qualitative data were collected concurrently. The quantitative and qualitative strands were related but independent, and their findings were reported in parallel without integration.

### 2.2 Setting

The study was conducted at the College of Medicine and Health Sciences (CMHS) at UAE University, the largest medical school in the country, which graduates approximately 100 physicians annually. At CMHS, in the first 2 years, students complete professionalism and healthcare principles courses while studying general education and foundational medical sciences. In years 3 and 4, they study basic and clinical sciences through an integrated, system-based curriculum and develop clinical assessment skills through simulation-based learning. Direct clinical exposure to patients primarily begins during the final 2 years through clinical clerkships. No formal professionalism training is offered after the first two years of basic sciences.

### 2.3 Eligibility criteria

Medical students studying at CMHS in 2023 in pre-clinical and clinical years (Years 1–6) who consented to participate in the study.

### 2.4 Study tools

Data collection for the study was conducted using the following instruments:

(a)    Demographic information, including age, sex, academic year, and intended medical specialty.
(b)    The Professionalism Assessment Scale (PAS) is a 22-item self-assessment tool designed to evaluate attitudes toward professionalism. This instrument measures professionalism through its three primary subcomponents–empathy, professional relationships, and responsibility–and has been demonstrated to be a valid and reliable measure of professionalism attitudes among medical students and residents (20).
(c)    Professional Identity Questionnaire (PIQ). The Professional Identity Questionnaire (PIQ) is a validated, quantitative tool for assessing professional identity, demonstrating both validity and reliability in measuring medical students’ professional identity (21).
(d)    To complement the quantitative data and provide contextual insight, an open-ended qualitative questionnaire was utilized to gather students’ perspectives on learning and assessing professionalism. Students were asked two key questions: “How do you learn professionalism?” and “How should professionalism be assessed?”

### 2.5 Study size

All 638 currently enrolled students, comprising the accessible study population, were invited to participate using a census sampling approach. As this was an exploratory study, no formal sample size calculation was performed. One hundred eight students (16.9%) consented and completed the questionnaires.

Students were grouped based on their curriculum stage into four groups for analysis, namely Basic Sciences (Years 1–2), Preclinical (Years 3–4), Early Clinical (Year 5), and Late Clinical (Year 6).

### 2.6 Data analysis

Data were analyzed using the IBM SPSS version 22. Both the PAS and PIQ were administered in English, following the original response formats, including reverse-scored items where applicable. After descriptive analysis, bivariate comparisons were made using Pearson’s correlation coefficient and one-way ANOVA tests. Reliability analysis was carried out using Cronbach’s alpha. Principal component analysis was used for factor analysis. Factors with eigenvalues greater than one were evaluated. Multivariate analysis used linear regression with PAS and PIQ as outcome variables. An alpha of 0.05 was considered the cut-off for statistical significance. Missing values were minimal.

The qualitative data underwent inductive thematic analysis, where initial codes were developed to capture key concepts and refined iteratively into cohesive themes. Two independent experts were involved to ensure reliability and reduce individual bias. Each expert reviewed and coded the data separately, followed by iterative discussions to resolve any discrepancies. Consensus was reached through discussion until complete agreement on the final themes was achieved. Subsequently, a matrix analysis was performed to determine the frequency of themes in each question.

### 2.7 Ethics approval

The Research and Graduate Studies Ethics Committee approved the study, United Arab Emirates University, Al Ain, UAE (Reference No: ERSC_2023_3069ERSC_2023_3069). Informed consent was obtained from participants regarding the publication of the findings.

## 3 Results

One hundred eight medical students out of 638 enrolled at the college completed validated study tools (response rate 16.9%). The mean age was 21.4 years, with a standard deviation (SD) of 2.5 (range: 17–35 years). The gender distribution reflected the underlying enrollment pattern: 71 (65.7%) were female participants, while 37 (34.3%) were male participants. Table 1 shows the demographic and baseline characteristics of the study participants.

**TABLE 1:** Demographic characteristics of study participants (*n* = 108 medical students).

Characteristics	Females	Males
	*N* (percent)	*N* (percent)
**Age**		
20 or less	17 (23.9)	12 (32.4)
21–22	38 (53.5)	18 (48.6)
23+	16 (22.5)	7 (18.9)
**Stage**		
Basic sciences	4 (5.6)	5 (13.5)
Preclinical	21 (29.6)	13 (35.1)
Early clinical	20 (28.2)	11 (29.7)
Late clinical	26 (36.6)	8 (21.6)
**Intended specialty type**		
Surgical	23 (32.4)	22 (59.5)
Medical	42 (59.2)	11 (29.7)
Non-clinical	4 (5.6)	2 (5.4)
Undecided	2 (2.8)	2 (5.4)

The group’s mean score on the Professionalism Assessment Scale (PAS) was 101.4 out of a total score of 110, and the overall score of the Professional Identity Questionnaire (PIQ) for the entire group was 38.5 out of 50. Among the three subscales of the PAS, students scored higher in Empathy (96%) compared to Responsibility and Professional Relationships (90% and 89%, respectively).

### 3.1 Reliability analysis

The reliability coefficient (Cronbach’s alpha) was 0.763 for the PAS questionnaire and 0.767 for the PIQ instrument. The reliability coefficients remained stable if any of the items were removed from the analysis, indicating internal consistency.

### 3.2 Factor analysis

The two questionnaires used Principal component analysis to discover underlying common factors. For the PAS and PIQ instruments, the Kaiser-Meyer-Olkin Measure of Sampling Adequacy was 0.656, indicating a likely utility of factor analysis. This was supported by a significant Bartlett’s Test of Sphericity (*p* < 0.001). The extraction communalities were greater than 0.5 for all items, indicating that all items fit with the factor solution, and none should be dropped from the analysis. The principal component analysis of PAS yielded seven factors with eigenvalues >1, accounting for 64% of the variability in the original items. The extracted and rotated factors had the same (64%) variance explained. Subscales were not extracted from the factors as a clear pattern did not emerge. The principal component analysis of PIQ yielded three factors with eigenvalues >1, accounting for 67% of the variability in the original items. The extracted and rotated factors had the same (67%) variance explained.

### 3.3 Bivariate correlations

PAS scores correlated strongly with empathy (*r* = 0.804, *p* < 0.001, *n* = 108), professional relationships (*r* = 0.804, *p* < 0.001), and Responsibility (*r* = 0.839, *p* = < 0.001). Empathy was associated with Professional Relationships (*r* = 0.510, *p* < 0.001) and Responsibility (*r* = 0.599, *p* < 0.001). Responsibility was moderately correlated with Professional Relationships (*r* = 0.613, *p* = < 0.001). Age did not correlate with scores on the PAS, PIQ, Empathy, Responsibility, and Professional Relationships (all *r* values < 0.2). Similarly, PIQ did not correlate with PAS, Empathy, Professional Relationships, or Responsibility (all *r* values < 0.14). Students interested in surgical specialties had lower scores on PAS but higher scores on professional identity. Students interested in non-clinical specialties had lower scores for Professional identity than other groups (Table 2), although these differences did not reach statistical significance.

**TABLE 2:** Comparison of professional scores in different domains.

Variables	Categories	PAS		PIQ		Empathy		Professional relationships		Responsibility	
		Mean	(SD)	Mean	(SD)	Mean	(SD)	Mean	(SD)	Mean	(SD)
Age groups	≤20	100.9	(6.7)	39.3	(6.3)	47.6	(2.4)	35.4	(3.5)	17.9	(1.9)
	21–22	103.3	(5.8)	38.1	(5.9)	48.8	(1.7)	36.2	(3.1)	18.2	(1.9)
	23+	102.2	(5.5)	39.4	(6.9)	47.6	(2.4)	36.4	(2.4)	18.2	(1.8)
Four stages in medical college	Basic science	96.8	(8.7)	37.7	(4.6)	46.1	(2.9)	33.0	(4.3)	17.7	(2.3)
	Preclinical	103.3	(4.4)	39.9	(6.2)	48.6	(1.6)	36.5	(2.2)	18.1	(1.8)
	Early clinical	102.1	(6.8)	38.6	(6.6)	48.6	(1.9)	35.7	(3.5)	17.9	(2.2)
	Late clinical	103.2	(5.3)	37.9	(6.3)	48.1	(2.4)	36.7	(2.6)	18.4	(1.6)
Intended Specialty Group	Medical	103.3	(5.0)	38.2	(6.6)	48.4	(2.0)	36.6	(2.4)	18.3	(1.7)
	Surgical	100.8	(6.8)	39.5	(6.1)	47.8	(2.3)	35.3	(3.5)	17.7	(2.0)
	Non-clinical	104.8	(6.3)	36.0	(5.0)	49.2	(1.0)	36.5	(4.5)	19.2	(1.2)
	Undecided	104.0	(6.7)	40.5	(1.9)	49.5	(1.0)	36.0	(3.9)	18.5	(1.9)

### 3.4 Comparison of means

**FIGURE 1:**
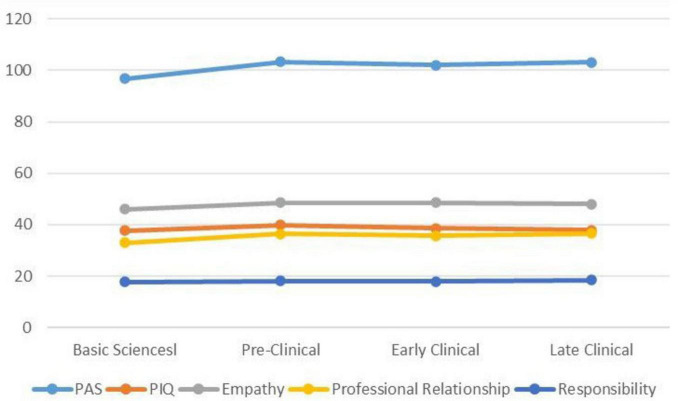
Professionalism scores across the four stages of medical student progression.

PAS (*p* = 0.024), Empathy (*p* = 0.009), Professional Relationships (*p* = 0.007), and age (*p* = < 0.001) were significantly different across the four stages of medical students’ progression. All these variables had the lowest scores in the Basic Sciences stage (Figure 1). However, the means of Responsibility (*p* = 0.604) and PIQ (*p* = 0.567) across the four stages were not significantly different. Mean scores in basic science were lower than other stages for PAS (p = 0.024), empathy (p < 0.001), and professionalism (p < 0.001) but not for PIQ (p = 0.56) (Table 2).

### 3.5 Multivariate analysis

On linear regression with PAS as the outcome, none of the explanatory variables (age, sex, intended specialty, stage of medical student progression) were statistically significant predictors (adjusted R-squared = 0.375).

### 3.6 Qualitative analysis

Qualitative analysis indicated that students learned professionalism through dedicated courses, reflection, observation of practice, and emulation of role models. Some viewed professionalism as an innate trait. Matrix analysis highlighted observation and experiential learning as prevalent modes, with less emphasis on self-reflection. Regarding professionalism, assessment, observation, and feedback-based methods were most common, while self-reflection and multisource evaluation were less cited. Tables 3, 4 present major themes and sub-themes based on frequency.

**TABLE 3:** Themes and subthemes of how students learn professionalism.

Themes	Frequency	Subthemes	Exemplars
Observation and experience	59	Clinical practice; peer learning; real-life encounters	“Most often by observation. I can observe the doctor’s behavior with patients and take from it both the negatives and positives. I apply the positives and avoid the negatives.”
Formal education and training	23	PPC course; lectures; self-study	“By studying the PPC course” “From lectures or classes given by faculty members”
Reflection and self-awareness	9	Learning from mistakes; self-awareness and morals	“….and by learning from my mistakes and others”
Innate traits and common sense	9	Upbringing; willingness; inherent nature	“professionalism comes naturally” “It is an innate thing.”
Modeling Behavior	8	Role modeling; Examples	“I model my behavior based on doctors and other professionals whom I see as exemplifying what it means to be a professional.”
Professional skill development	7	Time management; communication; ethics; continuous learning	“Focus on developing excellent communication skills, demonstrate empathy and respect patients, adhere to ethical standards, maintain confidentiality, update and develop medical knowledge and skills through practice.”

PPC, professionalism in practice course.

**TABLE 4:** Themes and subthemes of how professionalism should be assessed.

Themes	Frequency	Subthemes	Exemplars
Feedback-based evaluation	26	Supervisor & colleague feedback; patient feedback; team interactions	“Feedback from other colleagues and supervisors” “Should be assessed by giving the patient a questionnaire after each doctor visit, the questions should focus on various ethical aspects of medical practice.”
Observational evaluation	22	Communication observation; behavioral observation; clinical encounters	“The way the physician interacts with his or her patients and the rest of the medical team” “By observing how one deals with different situations and people”
Self-assessment and reflection	11	Reflective practice; self-assessment; feedback comparison	“…and scrutiny of self-assessments compared with assessments by others” “By reflecting on your patients’ and colleagues’ feedback”
Formal assessment	9	Knowledge testing; structured methods; quantitative scoring	“Case exam (assessing students based on their written response)” “Evaluation given by marks. Since we students care about our marks mostly”
Multisource evaluation	9	Comprehensive criteria; diverse methods	“…be assessed on multiple levels” “Professionalism assessment should take into account a doctor’s dress, manners, and tone as well as their capacity to handle difficult situations efficiently and navigate emotional situations……”

## 4 Discussion

Our study examined the scores of professionalism attributes and professional identity among medical students across their curriculum phases, utilizing the Professionalism Assessment Scale (PAS) and the Professional Identity Questionnaire (PIQ). Through Cronbach’s alpha and factor analysis, we confirmed the reliability and validity of the tools implemented in our study. Bivariate correlation analysis revealed a good correlation among the three attributes of PAS. However, the PIQ score did not show a correlation with any of the professionalism attributes.

There was an increase in PAS, empathy, and professional relationship scores from the basic sciences to the pre-clinical years, followed by a plateau during the clinical years. In contrast, responsibility and professional identity scores remained unchanged throughout all stages. The qualitative analysis of students’ open-ended comments on learning and assessment of professionalism provided additional insights into this phenomenon.

### 4.1 Correlations among professionalism constructs

Professionalism and professional identity are overlapping and mutually reinforcing; discussing professionalism through the lens of identity can deepen understanding and promote professional behavior (22). In our study, significant associations were found between PAS scores and the constructs of empathy, professional relationships, and responsibility. Bivariate analyses revealed strong correlations between empathy and professional relationships, as well as between empathy and responsibility. Responsibility, in turn, showed a moderate correlation with professional relationships.

### 4.2 Distinct nature of professionalism and identity

A recent cross-sectional study found a moderate positive correlation between medical students’ perceptions of their professional identity and their level of professionalism (23). However, our data showed that PIQ scores had low correlation with empathy, PAS, professional relationships, and responsibility (all *r* values < 0.14). Combined with the prolonged plateau in PIQ scores, our findings suggest that professional identity may be conceptually distinct from professionalism than previously assumed. Although prior literature has highlighted the two as distinct but interrelated constructs (5), our findings argue for separate, targeted strategies in curriculum design to address each area. This distinction warrants further exploration to refine professional development frameworks in medical education.

### 4.3 Comparative scores and gender trends

Our students’ overall professionalism and professional identity scores align with previous research. The mean professional identity score was 38.5 out of 50, mirroring results from a Dutch validation study (mean score: 38.7) (21). Similarly, the average professionalism score of 101.4 out of 110 was slightly higher than the 96.4 reported in a Turkish validation study (20). Despite a higher proportion of female participants in our study (66%) than in Tanrıverdi et al. (51%), we did not observe gender-based differences in empathy or professional relationship scores. This contrasts with earlier findings suggesting gender influences these domains (24), highlighting potential contextual or cultural factors that affect gender differences.

### 4.4 Progression through curriculum and plateau effect

A key finding was the increase in PAS, empathy, and professional relationships scores from basic sciences to pre-clinical years, followed by a plateau through clinical years. In contrast, responsibility and professional identity scores remained consistent across all stages. This pattern differs from a cross-sectional Irish study, which found random variations in professional identity across student cohorts without a clear trajectory (25). The observed increase in early years may be attributed to structured professionalism courses and coping with academic transitions, supporting existing evidence that such transitions can enhance identity and professionalism development (26).

The lack of significant change in scores during the pre-clinical to clinical transition indicates that neither formal instruction nor the hidden curriculum substantially impacts these constructs in later years. This finding is explained by the literature that highlights the benefits of longitudinal and early clinical exposure on professional identity development (27). A strong preexisting cultural and religious identity, coupled with a relative lack of experientially immersive curriculum, may also be contributing to the plateau. These results underline the importance of continuous and integrated approaches in medical curricula, especially in the UAE and similar educational contexts, to foster sustained growth in professionalism and identity.

### 4.5 Specialty preference and its influence

Students aspiring to surgical careers demonstrated slightly lower professionalism scores but higher professional identity scores, although these differences were not statistically significant. This aligns with a Danish multi-institutional study where surgical-track students had notably lower empathy scores (28). Similarly, students inclined toward non-clinical specialties in our study reported lower professional identity scores, reaffirming patterns seen in earlier studies (29). These insights may inform guidance and mentorship programs tailored to students’ career aspirations.

### 4.6 Student perspectives on learning and assessment

Qualitative responses highlighted two primary sources of professionalism development: formal education in the early years and role modeling during clinical exposure. However, consistently integrating these methods across all years remains challenging (30). Some students described professionalism as innate, and few explicitly mentioned reflection or self-awareness. The recurring emphasis on observation and role modeling underscores the hidden curriculum’s role, consistent with previous research findings (31, 32). In terms of assessment, students favored observational and multisource feedback, which aligns with the broader literature on best practices in professionalism assessment (33). These preferences should be considered when designing evaluation methods to ensure alignment with learner expectations and educational goals.

### 4.7 Limitations

The 17% participation rate and the study’s cross-sectional nature limit generalizability and preclude longitudinal analysis. However, despite this limitation, the stagnant scores of professional identity across clinical years highlight a significant finding of concern.

The study occurred at a medical college that predominantly admitted students of one nationality. While this may restrict the generalizability of the findings, it aligns with the demographic composition of many medical colleges globally.

## 5 Conclusion

Our study suggests that professionalism and professional identity should be addressed as related but distinct frameworks. Using validated tools, we found that professionalism attributes–such as empathy, responsibility, and professional relationships–are strongly connected and tend to improve early on. In contrast, professional identity follows a separate path and shows limited correlation with these traits. The plateau observed during clinical years suggests that current curricula may not sufficiently support continued growth in these areas. Students highlighted the roles of formal teaching and role modeling, but few mentioned reflection, indicating missed opportunities for deeper learning. Medical curricula must therefore adopt longitudinal strategies, including experiential learning and reflective practice throughout all years to support lasting development. Further research should explore how curriculum structure and career pathways influence these developmental patterns to inform targeted reforms.

## Data Availability

The raw data supporting the conclusions of this article will be made available by the authors, without undue reservation.
